# Effects of Maltodextrins on the Kinetics of Lycopene and Chlorogenic Acid Degradation in Dried Tomato

**DOI:** 10.3390/molecules24061042

**Published:** 2019-03-16

**Authors:** Pedapati S.C. Sri Harsha, Vera Lavelli

**Affiliations:** 1 Institute of Food and Health, University College Dublin, Dublin 4, Ireland; siva.pedapati@ucd.ie; 2 DeFENS, Department of Food, Environmental and Nutritional Sciences, Università degli Studi di Milano, via Celoria 2, 20133 Milano, Italy

**Keywords:** maltodextrins, lycopene, chlorogenic acid, water activity, kinetics

## Abstract

Maltodextrins (MD) are frequently used as processing aids in tomato drying. The aim of this study was to investigate the effect of the addition of MD on the stability of lycopene and chlorogenic acid, which are the main lipophilic and hydrophilic antioxidants in processed tomato, respectively. Tomato powder added with 10% MD (dextrose equivalents, DE 12) and a control tomato powder were stored in the water activity (a_w_) range 0.17–0.56, for 180 d at 30 °C. At the a_w_ level of 0.17, which was below the monolayer moisture content (*M*o), chlorogenic acid was stable, while lycopene content decreased faster in tomato added with MD than in control tomato, probably due to a decrease in matrix hydrophilicity and greater oxygen diffusion in the oil phase. Maximum stability occurred in both tomato powders at a_w_ of 0.3, that was in close proximity to *M*o (first-order rate constant for lycopene, k = 7.0 × 10^−3^ d^−1^ in tomato added with MD). At high a_w_ levels, MD increased the rate of lycopene degradation with respect to the control, possibly by hampering its regeneration by chlorogenic acid, which conversely was found to be more stable than in the control tomato.

## 1. Introduction

It is well established that the consumption of tomato is associated with various health benefits ranging from cardiovascular health to protection against cancers [1,2,3]. These health properties have been proven in a number of epidemiological studies [4,5] and in vitro model systems. The mechanisms underlying tomato health effects depend mainly on lycopene, which is its peculiar lipophilic antioxidant. Lycopene is a highly unsaturated acyclic carotenoid, which exhibits a high physical quenching rate of singlet oxygen [6]. Protective effects of lycopene against oxidative stress have been reported in a number of studies [7,8]. Moreover, tomatoes are a source of both ascorbic acid and phenolic compounds, mainly chlorogenic acid, which are common hydrophilic antioxidants among vegetables, which are also regarded as important bioactive compounds [9,10,11].

Since humans cannot synthesize these compounds *de novo*, and considering the beneficial health effects they impart, their content in raw and processed tomato has become an important area of research. Lycopene is stable upon thermal processing of tomato. Moreover, the thermal processes applied to tomato can result in the breakdown of cell walls, thus improving lycopene bioaccessibility. Indeed, numerous studies have shown that lycopene bioavailability is higher for processed tomato than for fresh tomato [12]. Conversely, ascorbic acid is very sensitive to thermal degradation, while the content of phenolic compounds in tomato increases after thermal treatments, due to solubilization from the plant cellular wall as well as inactivation of polyphenol oxidase [10].

One of the main technologies applied to tomato is drying. Lycopene and chlorogenic acid are not affected by drying but their stability is low during storage of the dehydrated product [13]. Maltodextrins (MDs) are frequently used as processing aids in tomato drying. MDs are among the most important carrier agents in the spray-drying process, mainly because they form low viscosity solutions in high concentrations that can be efficiently spray-dried [14]. MDs with DE 6–21 were applied in the spray-drying of tomatoes, resulting in increased efficiency of the process due to the MD ability to encapsulate low molecular weight sugars and reduce wall deposition problems [15]. Moreover, MDs are used for tomato puree foam drying [16].

MDs are also applied as osmotic agents in osmotic dehydration, which results in the development of intermediate moisture products having a lower water activity (a_w_) imparted by solute gain and water loss. Osmotic dehydration is performed as a pre-drying treatment at low temperatures, because it is a less energy-intensive process than other drying processes [17]. Following this latter approach, a solution of 27.5% MD with DE 10 and 10.0% NaCl, combined with the application of a vacuum pulse (100 mbar, 20 min) was used at 40 °C for the osmotic dehydration of tomatoes. The use of MD decreased NaCl incorporation and increased the effective diffusivity of water, thus accelerating the osmotic dehydration process [18]. Moreover, MDs have been applied as encapsulating agents for phenolic compounds, providing protection against oxidative degradation during processing and storage of foods [19].

Despite the common use of MDs in various drying technologies, little information is available on the effect of MDs on the stability of the main antioxidants of tomato. Previous studies revealed low stability of lycopene in starch or MDs added matrices. In fact, soluble starch was used as a carrier for the encapsulation of lycopene from *Rosa rubiginosa* by spray-drying, resulting in a half-life of 5 d at 21 °C [20]. The peel fraction of tomato mixed with MD (DE not specified) before spray-drying showed a half-life of 12 d [21]. In this latter study a control matrix without MD was not run in parallel, thus the specific effect of MD on lycopene stability cannot be derived. It is worth noting that the kinetics of lycopene degradation in dried matrices is greatly affected by the water activity (a_w_) level [13,22]. Moreover, the presence of MDs affects the water sorption properties and the stability of dry foods [23]. Hence, the identification of the optimal a_w_ level to achieve upon drying is crucial to extend storage stability.

As food supply chains are constantly evolving toward healthier food production and better resource management, food technologies should combine the production of foods with promising bioactive compounds with sustainable processes [24]. From a sustainability perspective, drying, which is notably an energy-consuming operation, should be followed by storage at ambient temperature which prevents the need for energy-consuming cold storage facilities.

Therefore, the aim of this study was to set kinetic models for lycopene degradation in dried tomato matrices added with MD (DE 12) and to find the a_w_ conditions for maximum storage stability. The degradation reactions occurring in both the polar and the oil phases of the tomato matrix were investigated and a model for oxidative phenomena that can be useful for the stability optimization of lycopene-rich food matrix was proposed.

## 2. Results and Discussion

### 2.1. Matrix Composition and Hygroscopicity

**Figure 1:**
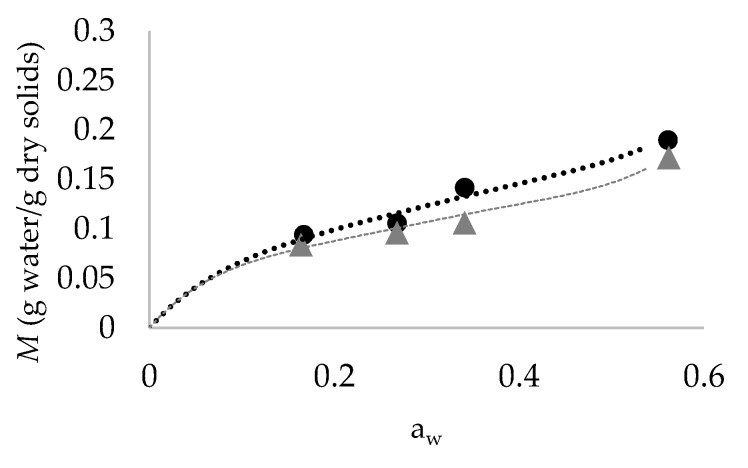
Equilibrium moisture content (*M*) as a function of water activity (a_w_) for tomato pulp powder (black line and symbols) and tomato pulp powder added with maltodextrins (MD) (grey line and symbols). Full symbols represent experimental data for samples equilibrated over saturated salt solution in a desiccator at 30 °C, lines represent adsorption isotherm obtained by fitting experimental data with the Guggenheim–Anderson–de Boer (GAB) model.

The composition of tomato pulp powder is shown in Table 1 and moisture isotherms of tomato products, fitted by the Guggenheim–Anderson–de Boer (GAB) equation, are shown in Figure 1.

The tomato pulp powder isotherm was best described as a Brunauer type II isotherm, which is associated with relatively strong interactions between absorbent and absorbate, and results from multi-layer absorption of water, capillary filling, and capillary condensation [25]. Sugars and organic acids present in tomato pulp powder likely contribute to its hygroscopicity, indeed tomato skin powder, which possesses more fiber and waxy cuticle materials is less hygroscopic than the tomato pulp powder [25].

As expected, the addition of 10% MD slightly decreased tomato hygroscopicity, since at a given a_w_ the amount of water adsorbed was always lower for tomato pulp powder added with MD than for tomato pulp powder (Figure 1). *M*o, *C* and *K* values were found to be 0.120 g water/g dry solids, 13 and 0.76 for tomato pulp powder and 0.099, 18 and 0.86 for tomato pulp powder added with MD, respectively. *M*o corresponded to an a_w_ level of 0.28 for tomato pulp powder and 0.26 for tomato pulp powder added with MD. A slightly higher *M*o value for tomato pulp powder was observed by Xu et al. [25], i.e., 0.16 g waters/g dry solids.

### 2.2. Kinetics of Lycopene Degradation

**Figure 2:**
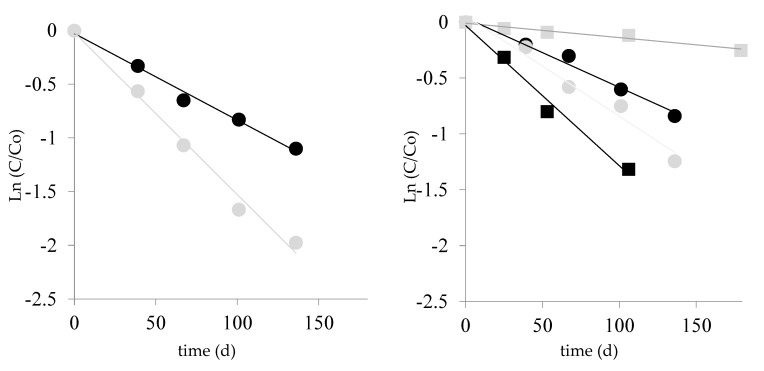
Time course of lycopene (circle) and chlorogenic acid (square) degradation in tomato pulp powder (black line and symbols) and tomato pulp powder added with MD (grey line and symbols) during storage at a_w_ 0.17 (on the left) and 0.56 (on the right). Symbols represent experimental data; lines represent fitting experimental data with first-order kinetics.

During storage, lycopene content decreased in both tomato pulp powder and tomato pulp powder added with MD by following first-order kinetics (Figure 2, Table 2).

Carotenoid degradation has been reported to follow radical-based reactions. These reactions could be initiated by transition metal ions. In fact, electron transfer occurs between transition metal ions like ferric iron and a carotenoid compound, forming the ferrous ion and the carotenoid radical cation that can start radical reactions, leading to further carotenoid loss [26]. Since lycopene is lipid soluble, its degradation could also be triggered by the autoxidation of unsaturated fatty acids, which in turn could be initiated by transition metal ions. In the presence of lipid alkyl, alkoxy and peroxy radicals, three types of reactions can involve the carotenoid compounds, namely: electron transfer reactions to form radical cations, reaction with radicals through hydrogen abstraction, or adduct formation reactions [26].

In freeze-dried tomato pulp powder, initial lycopene content was 2465 mg/kg on dry weight basis (d.w.) (Table 2). The degradation rate for lycopene in the pulp powder decreased with increasing moisture content, with half-lives at 30 °C increasing from 86 to 119 d when the a_w_ level increased from 0.17 to 0.32. The addition of MD to tomato pulp powder greatly decreased lycopene stability, especially at the lowest a_w_ of 0.17, where the half-life was 46 d, half than that observed in the absence of MD. Similarly, lycopene stability in tomato peel was very low at the a_w_ of 0.17, with a half-life of 41 d [13].

In previous studies, encapsulation of the lipid matrix containing lycopene was proposed to improve lycopene stability. Use of soluble starch and MD as a carrier before spray-drying of lycopene oleoresin and tomato peel, resulted in a half-life of 5 d and 12 d at 21–25 °C [20,21], respectively. The fast degradation rate of lycopene was also found by Chiu et al. [27] for the freeze-dried extract of tomato pomace obtained by supercritical carbon dioxide encapsulated in poly-γ-glutamic acid (a_w_ not specified), resulting in a half-life of 25 d at 30 °C. Tomato oleoresin was also encapsulated in zein by spray-drying. By this approach, the stability was even lower, resulting in a half-life of 10.9 d at 25 °C [28].

Hence, none of the hydrophilic polysaccharides or proteins proposed as a lycopene encapsulating agent was effective in increasing lycopene stability during storage. It may also be concluded that lycopene was not effectively surrounded by the hydrophilic polymers. In fact, only chemical modification of starch with the incorporation of a lipophilic compound was found as a good encapsulation agent for lycopene, with retention higher than 50% after storage for 78 d at 25 °C [29].

### 2.3. Kinetics of Chlorogenic Acid Degradation

At a_w_ levels <0.32 no changes were observed in the content of chlorogenic acid of tomato products. Conversely, at a_w_ 0.56 chlorogenic acid content of tomato pulp powder decreased greatly, by following first-order kinetics with a half-life of 58 d (Figure 2, Table 3). It was previously observed that the rate of degradation of chlorogenic acid increases with an increase in a_w_ at values ≥0.55. This finding was related to an increase in molecular mobility, as assessed by ^1^H NMR [30].

The half-life of chlorogenic acid in apple pulp powder stored at a_w_ 0.54 at 30 °C was found to be 347 d [30], while in low-methoxyl-pectin (LMP) film stored at a_w_ 0.58 at 20 °C it was found to be 186 d [31]. Apple pulp powder has a low content of fat (0.46% d.w.), [30]. The lower stability of chlorogenic acid found in tomato pulp powder with respect to apple pulp powder could be attributed to its higher level in fats (2.0% d.w.), which can trigger radical-based reactions.

The presence of MD in tomato pulp powder was associated with significantly enhanced stability of chlorogenic acid, which showed a half-life of 533 (Table 3). MDs have been reported to encapsulate chlorogenic acid [32]. This could be the reason for their protecting effect.

### 2.4. Overview of Degradation Phenomena in the Water and Oil Compartments

Based on the results obtained, a model of oxidation phenomena occurring in both the polar and the oil phases of tomato pulp powder was developed.

**Figure 3:**
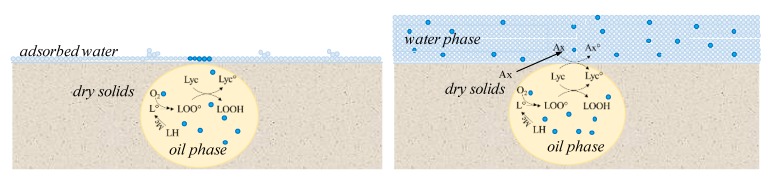
Model for oxidative reactions in dry tomato powder at a_w_ below the monolayer moisture (*M*o) (left side) and at a_w_ well above *M*o (right side). 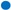 = oxygen, 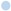 = water; Lyc = lycopene, Ax = hydrophilic antioxidant, LH = unsaturated fatty acid, L° and LOO° = radicals, LOOH = peroxide. At a_w_ below *M*o, MD could enhance oxygen binding due to decreased hydrophilicity of the matrix and larger pore sizes, thus increasing lycopene degradation rate. At a_w_ well above *M*o, MD could encapsulate the hydrophilic antioxidants thus hampering lycopene regeneration.

As a general rule, at a_w_ of 0.17 no changes were observed in the polar antioxidant content—i.e., chlorogenic acid—while the lowest lycopene stability was observed. At this a_w_ level, moisture content was below *M*o. Since tomato pulp powder is a heterogeneous matrix, below *M*o, water molecules are bound to the most hydrophilic sites of the dried solids. Under these conditions, water molecules are likely in a monolayer arrangement, but clustering of water molecules is also possible when the affinity of water towards dry solids is lower relative to cohesion between water molecules (Figure 3). Whatever the arrangement of water molecules, below *M*o, part of the dry solid surface is directly exposed to air. As for moisture, oxygen sorption occurs on the dry solids [33]. It is likely that preferential sites for oxygen sorption are the most hydrophobic sites of the dry solids, where moisture is excluded. Moreover, the dry solids can contain a different amount of void volumes, depending on the drying process. According to Prado et al. [34], at low water content, the nonpolar carotenoid degradation rate is enhanced by the porosity of the matrix. In this hypothesis, Harnkarnsujarit et al. [35] studied the microstructure of MD and sugar matrices in freeze-dried systems by scanning electron microscopy and demonstrated that freezing of MD without sugars formed larger pore sizes in the freeze-dried solids than in the presence of sugar (glucose, fructose, and sucrose). Additionally, MDs were proven to be a very good encapsulating agent for low molecular weight sugars such as fructose and organic acids [36]. It may be hypothesized that addition of MD to tomato pulp powder caused the formation of larger pore sizes during freeze-drying and decrease the hydrophilicity of the matrix due to sugar encapsulation, thus favoring oxygen binding. This could explain the decrease in lycopene stability upon MD addition to tomato. On the contrary, the presence of low-molecular-weight sugars in tomato pulp powder without MD was crucial to increase lycopene stability.

At the a_w_ levels of 0.22 and 0.32 the rate for lycopene degradation decreased. These a_w_ levels are in close proximity to the monomolecular moisture content. The decrease in degradation rate could be due to the small amount of water that combines metal ions and precipitate them as insoluble hydroxides [13]. Additionally, water covers the solid surface. This means that oxygen molecules have to diffuse through the layers of water clusters first, before they can come into contact with the sorption sites on solids’ surface. Water is a poor solvent for oxygen [33]. Indeed, oxygen solubility is of 8–10 mg/L [37]. Consequently, the amount of oxygen absorbed to the matrix decreases with respect to that absorbed at a_w_ 0.17.

At a_w_ 0.56, the stability decreased in the hydrophilic compartment of the tomato pulp powder as shown by the increased degradation rate of chlorogenic acid. Conversely, at a_w_ levels below 0.32, the presence of the hydrophilic antioxidant chlorogenic acid resulted in having no influence on lycopene degradation. In fact, while lycopene degradation was maximum at a_w_ of 0.17, no change occurred in chlorogenic acid content at a_w_ < 0.32. The decrease of chlorogenic acid stability at a 0.55 could be due to the increase in molecular mobility in the water phase, which favors the diffusion of both the oxygen molecules and the oxygen-sensitive targets. Oxygen solubility in oil was reported to be 47 mg/L at 30 °C [37], which is higher than in water. Indeed, vegetable oils solubilize around 4 to 5 times more oxygen than water does, depending on the temperature [38]. On the other hand, the rate for chlorogenic acid degradation in tomato pulp powder at a_w_ 0.55 was much higher than those reported for other matrices with lower or no fat content [30,31]. Moreover, while chlorogenic acid degradation rate increased in tomato pulp powder, the rate of lycopene degradation in the lipid compartment did not increase. It may be hypothesized that an active interaction of antioxidants occurred within and across the hydrophilic and lipophilic compartments of the tomato matrix, as already observed in various model systems [39]. Through this interaction, lipid peroxy and alkoxy radicals and/or lycopene radicals are regenerated by hydrophilic antioxidants such as chlorogenic acid (Figure 3). Indeed, chlorogenic acid was found to be a very efficient scavenger of the radicals formed during lipid peroxidation [40]. However, in the presence of MD the hydrophilic antioxidants were most likely encapsulated in this polymer and could not exert free-radical chain-breaking antioxidant activity, consistent with higher stability of chlorogenic acid but lower stability of lycopene.

## 3. Materials and Methods

### 3.1. Preparation of Tomato Pulp Powder

Tomato fruits (about 30 kg) were washed with water and heated for 60 min at 100 °C and homogenized with a food processor for 2 min at maximum speed and then refined using a screw extractor (model 9008 Reber, Luzzara, Italy). The pulp powder was recovered and the pomace (peels and seeds) discarded. The pulp powder was separated into two aliquots. One of these was added with 10% (on dry weight basis, d.w.) of MD D12 and then homogenized with Ultra-Turrax (T25 Janke & Kunkel IKA Labortechnik) for 2 min. The samples were immediately placed on aluminium trays and frozen at –45 for 16 h. Then the samples were freeze-dried (Lyoflex Edwards, Crawley, UK) by applying three subsequent time-temperature steps, namely: −45 °C for 8 h, −20 °C for 24 h, 0 °C for 24 h, and 10 °C for 10 h. The chamber pressure was maintained at 30 Pa throughout the drying process. Then, the powders were ground and sieved (800 μm). The other aliquot did not have MD added. Samples were immediately freeze-dried (Lyoflex Edwards, Crawley, UK) 

Freeze-drying was performed at −45 °C for 8 h, −20 °C for 24 h, 0 °C for 24 h, and 10 °C for 10 h. The chamber pressure was maintained at 30 Pa throughout the drying process. Then, the powders were ground and sieved (800 μm).

### 3.2. Storage Study

Tomato powders were placed into Petri dishes (6 cm diameter, 5.5 g of product in each dish) positioned inside airtight plastic chambers on wire-mesh racks situated above saturated salt solutions. The chambers were stored for 6 months at 30 °C. To create different environments, the following saturated salt solutions were used: LiCl (a_w_ = 0.113 ± 0.002), CH_3_COOK (a_w_ = 0.216 ± 0.005), MgCl_2_ (a_w_ = 0.324 ± 0.002) and NaBr (a_w_ = 0.560 ± 0.004). Duplicate chambers were incubated for each a_w_ level. The moisture content and a_w_ after freeze-drying were 8.6 g/100g and 0.17, respectively, with no significant differences between the tomato powders. The samples stored at a_w_ 0.113 maintained their initial a_w_ (0.17) during storage, while all the other samples reached the a_w_ level of the saturated solution in 5 days.

### 3.3. Moisture Content and a_w_

The moisture content of the tomato powders equilibrated at the various relative humidity conditions was determined using a vacuum oven at 70 °C and 50 Torr for 18 h. The a_w_ of tomato powders and saturated salt solutions was checked using a dew point hygrometer (Aqualab, Decagon Devices, Pullman, WA, USA). Duplicate determinations were made for each sample.

Moisture isotherms were developed for the tomato pulp powder and tomato pulp powder added with MD by plotting the equilibrium moisture content (M) versus the storage a_w_. The Guggenheim–Anderson–de Boer (GAB) Equation (1) was used to fit the experimental data:

(1) $$M=\frac{M_{o}CKa_{w}}{\left(1-Ka_{w})(1-Ka_{w}+CKa_{w}\right)}$$

where *M* is the equilibrium moisture content on a dry basis (g of water/g of dry solids); *M*_o_ is the monolayer moisture content on a dry basis; *C* and *K* are constants [13].

### 3.4. Titratable Acidity

Freeze-dried tomato powders were diluted with water (0.5 g of powder in 20 mL, in duplicate). Titratable acidity was determined by titration with 0.1 M NaOH to pH 8.1. Results were expressed as grams of citric acid per 100 g of dry product.

### 3.5. Soluble and Insoluble Fiber, Protein, Fat, and Ash

Fiber, protein, fat, glucose, fructose and ash values measured according to AOAC Official Methods of Analysis [41]. Duplicate determinations were made, and results are expressed as milligrams per kilogram of fresh product (f.w.)

### 3.6. HPLC Equipment

The HPLC equipment consisted of a model 600 HPLC pump coupled with a Waters model 2996 photodiode array detector, operated by Empower software (Waters, Vimodrone, Italy).

### 3.7. Lycopene

Tomato powders were analyzed monthly up to 139 d. A two-step extraction was applied using tetrahydrofuran (THF) stabilized by the addition of 0.1% butylated hydroxytoluene (2,6-di-tert-butyl-*p*-cresol, BHT). Aliquots of tomato powders (0.125 g d.w.) were added to 10 mL of stabilized THF. The mixture was vortexed for 1 min and centrifuged (12.000× *g* at 5 °C for 10 min). The supernatant was recovered into a 25 mL flask. Ten milliliters of stabilized THF was added to the residual solids. The mixture was vortexed for 1 min, stirred for 30 min with a magnetic stirrer, and then centrifuged (12.000× *g* at 5 °C for 10 min).

The extracts were pooled and brought up to 25 mL with stabilized THF. Extractions were carried out in triplicate. Lycopene content was analyzed by HPLC as described previously [13]. In brief, a Vydac 201TP54 C18 column (250 × 4.6 mm i.d., 5 μm particle size), equipped with a C18 precolumn, was used (Labservice Analytica, Anzola dell’Emilia, Italy). Chromatographic separation was performed with methanol/stabilized THF (95:5) as an eluent under isocratic conditions, 1.0 mL/min flow rate, at room temperature. Peaks were detected at 454 nm. Lycopene was quantified from a calibration curve using a pure standard and expressed as milligrams per kilogram of dry product.

### 3.8. Chlorogenic Acid

Aliquots of tomato powders (0.25 g dw) were analyzed monthly up to 180 d, except for samples incubated at aw 0.56, which were analyzed up to 106 d. Extraction was performed with 10 mL of methanol. The mixture was vortexed for 1 min, mixed continuously for 30 min with a magnetic stirrer, and then centrifuged at 12.000× *g* and 5 °C for 10 min. Extractions were carried out in triplicate on initial samples and in duplicate for samples stored at different a_w_ levels. The phenolic contents of methanolic extracts were analyzed by HPLC as described previously [13]. A 250 × 4.6 mm i.d., 5 μm particle size, Symmetry reverse phase C-18 column (Waters) equipped with a Symmetry C-18 precolumn was used. Formic acid (5%) was added to both methanol and water before the following mobile phases were prepared: (A) water/methanol (95:5, *v/v*); (B) water/methanol (88:12, *v/v*); (C) water/methanol (20:80, *v/v*); (D) and methanol. The following gradient elution was used: 0−5 min, 100% A; 5−10 min linear gradient to reach 100% B; 10−13 min, 100% B; 13−35 min linear gradient to reach 75% B and 25% C; 35−50 min linear gradient to reach 50% B and 50% C; 50−52 min linear gradient to reach 100% C; 52−57 min, 100% C; 57−60 min, 100% D. The flow rate was 1 mL/min. Chlorogenic acid was quantified at 330 from calibration curves using pure standard and expressed as milligrams per kilogram of dry product.

### 3.9. Statistical Analysis

Data were processed using Statgraphics 5.1 (STCC Inc., Rockville, MD, USA). ANOVA, followed by Fisher’s least significant difference test (LSD *p* ≤ 0.05), was used.

## 4. Conclusions

In conclusion, the addition of MD to tomato pulp powder greatly enhanced the effect of a_w_ on lycopene stability. At a low a_w_ level below *M*o—i.e., when absorption sites were available on the solid surface—addition of MD decreased lycopene stability probably due to a decrease in matrix hydrophilicity and increase in oxygen binding. At high a_w_ levels—i.e., when water covered all the solid surface—MD decreased lycopene stability probably due to encapsulation of the hydrophilic antioxidants, such as chlorogenic acid, thus limiting their ability to regenerate lycopene. Maximum stability occurred at an a_w_ of 0.3, that is in close proximity to *M*o. Hence, control of a_w_ of dry tomato pulp powder with MD added can be a strategy to extend lycopene stability at ambient temperature.

## Figures and Tables

**Table 1:** Major components of tomato pulp powder (g/100 fresh weight.)

Quality Index	Percent Content
moisture	8.6 ± 0.2
protein	8.8 ± 0.9
fat	1.9 ± 0.2
insoluble dietary fiber	15.7 ± 1.2
soluble dietary fiber	2.7 ± 0.4
glucose + fructose	34.0 ± 1.6
ash	6.4 ± 0.5
titratable acidity	0.20 ± 0.01

**Table 2:** Initial concentration (mg/kg dry weight), storage temperature (°C), time (d), a_w_, first-order rate constant (d^−1^) and half-life (d) of lycopene in various matrices.

Matrix	Co	T	t	a_w_	k × 10^3^	t_1/2_	Ref.
Tomato peel, freeze-dried	7390 ± 70	30	139	0.17	19 ± 1.2	41	Lavelli et al. [13]
				0.22	10 ± 1.5	63	
				0.32	9.0 ± 1.1	81	
				0.56	5.0 ± 0.8	115	
Tomato concentrate + MD, spray-dried	494 ± 10	25	28	nd	57.6	12	Souza et al. [21]
Tomato pomace extract + poly-γ-glutamic, freeze-dried	134.2 ± 2.3	35	30	nd	24.7	28	Chiu et al. [27]
Tomato oleoresin + zein, spray-dried	nd	25	18	nd	63.6	10.9	Xue et al. [28]
Lycopene in oil +modified starch, spray-dried	5000	25	78	nd	nd	>78	Rocha et al. [29]
Tomato pulp powder, freeze-dried	2465 ± 20	30	139	0.17	8.1 ^cd^ ± 0.5	86	^1^ This study
				0.22	7.7 ^cd^ ± 0.5	90	
				0.32	5.8 ^a^ ± 0.2	119	
				0.56	6.2 ^ab^ ± 0.3	111	
Tomato pulp powder + MD, freeze-dried	2184 ± 20	30	139	0.17	15.0 ^e^ ± 0.8	46	^1^ This study
				0.22	7.0 ^bc^ ± 0.9	99	
				0.32	7.6 ^c^ ± 0.6	91	
				0.56	8.8 ^d^ ± 1.2	79	

^1^ Different letters (a–e) indicate significant differences among first-order rate constants (LSD, *p* < 0.05).

**Table 3:** Initial concentration (mg/kg dry weight), storage temperature (°C), time (d), a_w_, first-order rate constant (d^−1^) and half-life (d) of chlorogenic acid in various matrices.

Matrix	Co	T	t	a_w_	k × 10^3^	t_1/2_	Ref.
Apple pulp powder	1050 ± 20	30	30	0.56	2.0	347	Lavelli et al. [30]
Low-methoxyl-pectin film	10.0 ± 0.6	25	215	0.58	3.7 ± 0.6	186	Basanta et al. [31]
Tomato pulp powder, freeze-dried	104 ± 18	30	106	0.17	n.s.		This study
				0.22	n.s.		
				0.32	n.s.		
				0.56	12 ± 1	58	
Tomato pulp powder +MD, freeze-dried	171 ± 3	30	106	0.17	n.s.		This study
				0.22	n.s.		
				0.32	n.s.		
				0.56	1.3 ± 0.2	533	
